# Exploration of the antiviral mechanism of gypsum-licorice compatibility pairing in Ma-Xing-Shi-Gan decoction from the perspective of metal-organic supramolecular interactions

**DOI:** 10.3389/fmedt.2025.1651390

**Published:** 2025-09-01

**Authors:** Chang Lu, Ying Ma, Haoyu Zhao, Yu Zheng, Qun Zhao, Lili Lin, XiuZhen Chen, Linwei Chen, Gang Li, Rui Chen

**Affiliations:** ^1^ School of Pharmacy, Nanjing University of Chinese Medicine, Nanjing, China; ^2^ Department of Pharmacy, The Affiliated Taizhou People’s Hospital of Nanjing Medical University, Taizhou, China; ^3^ Jiangsu Province Engineering Research Center of Classical Prescription, Nanjing University of Chinese Medicine, Nanjing, China; ^4^ Jiangsu Key Laboratory of Pediatric Respiratory Disease, Institute of Pediatrics, Medical Metabolomics Center, Nanjing University of Chinese Medicine, Nanjing, China; ^5^ Clinical Research Center, The Second Hospital of Nanjing, Affiliated to Nanjing University of Chinese Medicine, Nanjing, China; ^6^ School of Artificial Intelligence and Information Technology, Nanjing University of Chinese Medicine, Nanjing, Jiangsu, China; ^7^ Jiangsu Province Engineering Research Center of TCM Intelligence Health Service, Nanjing University of Chinese Medicine, Nanjing, China

**Keywords:** licorice, gypsum, antiviral, compatibility, metal-organic supramolecule

## Abstract

The efficacy of the gypsum-licorice (SG-GC) pair was evaluated through its *in vivo* and *in vitro* anti-respiratory syncytial virus (RSV) activity. The results showed that SG-GC had significant efficacy against RSV infection in mice, which was close to that of the whole formula, and could significantly reduce the viral load in the lungs, improve the symptoms of lung injury, and reduce the inflammatory cell infiltration in the pathological sites. The decoction of single-flavored gypsum showed some anti-RSV efficacy, but not significant; when combined with sub-inhibitory concentrations of GA, it showed a significantly enhanced anti-RSV effect. The experimental results of this study suggest that gypsum may be a potential key antiviral substance, but its efficacy needs to be complemented by licorice; the significant enhancement of antiviral efficacy of SG-GC may be related to the formation of metal-organic supramolecules after the interaction of trace metal ions in gypsum and GA in licorice.

## 1 Introduction

The medicinal pair of gypsum-licorice (SG-GC) exemplifies a classic sovereign-courier compatibility, evaluated by Mr. Zhang Xichun, the renowned Chinese physician, who noted that “the combination of the two can drive away evil without harming good”. A review of the “Dictionary of Traditional Chinese Medicine Prescriptions” and the “Treatise on Febrile and Miscellaneous Diseases” indicates that licorice is the most frequently paired Chinese medicine with gypsum. Utilizing the sweet and soothing properties of licorice can help reduce phlegm and relieve coughs, while also moderating the cold nature of gypsum. Therefore, SG-GC is a classic medicinal pair in traditional Chinese medicine (TCM), widely used in formulas to clear heat, resolve phlegm, and alleviate coughs associated with lung heat (1). Among these formulas, Ma-Xing-Shi-Gan Decoction (MXSGT)—originating from Zhang Zhongjing's Treatise on Febrile and Miscellaneous Diseases—stands out as a representative prescription where SG-GC plays a pivotal role. This study focuses on decoding the antiviral mechanism of the SG-GC pair, with particular emphasis on its activity against respiratory syncytial virus (RSV).

MXSGT is well-known for its straightforward compatibility and impressive therapeutic effects. The scientific implications of its compatibility have consistently attracted the attention of the TCM community. This decoction comprises whole Ephedra (*Ephedra sinica* herb, Sovereign, 9 g), gypsum (Sovereign, 24 g), bitter almond (*Prunus armeniaca* seeds, minister, 9 g) and licorice (*Glycyrrhiza uralensis* roots, courier, 6 g). With its four key ingredients, MXSGT is recognized for its capacity to release heat, cool the body, clear the lungs, and alleviate asthma (2). In the clinical practice of TCM, MXSGT and its modified formulations are extensively employed to address pneumonia caused by various viral infections, including RSV, influenza A, and COVID−19 in pediatric patients. Throughout the COVID-19 pandemic, the “three prescriptions and three medicines” have demonstrated efficacy in combating viral infections and alleviating symptoms in numerous clinical cases. Notably, Qingfei Paidu Decoction, Huashi Baidu Decoction, Xuanfei Baidu Decoction, Lianhua Qingwen Capsules and Jinhua Qinggan Granules are all derived from MXSGT, further substantiating its effectiveness as an antiviral prescription (3).

Since the Han and Tang Dynasties, physicians have frequently utilized gypsum in medicine to clear heat, purge fire, and alleviate heat-related ailments. Analyzing the formulation of MXSGT reveals a significant reliance on gypsum, particularly when symptoms such as sweating, asthma, and lung heat are pronounced; in these instances, the quantity of gypsum may reach five times that of ephedra (4). The primary component of gypsum is calcium sulfate (CaSO_4_), an inorganic salt that exhibits slight solubility in water. This solubility results in a rapid attainment of saturation in aqueous decoctions, with the calcium concentration remaining constant despite increases in the quantity of gypsum. Additionally, gypsum contains various trace elements, including iron, zinc, and copper (5).

Increasing the amount of gypsum can significantly enhance the concentration of trace elements in the decoction, suggesting that trace metal ions may play a crucial role in the functional properties of gypsum. Similarly, some studies have indicated that the use of pure CaSO_4_, as opposed to gypsum, results in a lack of antipyretic effect, which may be attributed to the differences in trace metal content. Specific trace elements found in gypsum have been shown to possess antiviral properties. For instance, zinc has the ability to inhibit RNA viruses, including the dengue virus and the novel coronavirus (6, 7); manganese is involved in the activation of innate immunity, which helps to inhibit the activity of DNA viruses (8); and copper can generate reactive oxygen species, utilizing redox signaling mechanisms to eliminate viruses (9). These findings underscore the importance of considering trace elements in the study of gypsum and its compound formulas. They may provide insights into the differences in the medicinal properties of gypsum compared to CaSO_4_, or they may offer a novel approach to understanding the antiviral efficacy mechanisms of MXSGT.

Licorice is a well-documented antiviral agent, with its principal component, glycyrrhizinic acid (GA), demonstrating inhibitory effects against various DNA and RNA viruses (10). GA has been shown to inhibit the novel coronavirus (SARS-CoV-2), hepatitis B virus (HBV), and human papillomavirus (HPV), primarily by blocking viral attachment or enhancing host cell activity (11–13). The carboxyl and hydroxyl groups present in the GA structure readily form coordination bonds with metal ions, leading to the creation of metal-organic supramolecular complexes. A study utilizing high-performance liquid chromatography (HPLC) indicated that the addition of gypsum affects the concentrations of other key components in MXSGT, notably resulting in a decrease in GA content. This decrease is hypothesized to arise from the formation of numerous metal-GA supramolecular complexes during the co-cooking process. The formation of these metal-organic supramolecules may enhance the activity of the original organic components or generate new pharmacological properties, particularly in antiviral applications. For instance, polyphenol-zinc complexes have been shown to significantly reduce genome replication levels across various respiratory RNA virus groups, including human coronavirus OC43 (HCoV-OC43), influenza A virus (IAV), and human metapneumovirus (hMPV) (14); similarly, copper complexes derived from pine resin ligands can effectively inactivate the novel coronavirus (SARS-CoV-2) (15). Therefore, it is imperative to conduct comprehensive research on the antiviral efficacy of metal-GA supramolecules in MXSGT.

RSV is a globally prevalent respiratory virus that primarily affects infants, the elderly, and individuals with compromised immune systems, and it is a leading cause of pneumonia in young children. Currently, there is a scarcity of effective RSV vaccines (16), and no specific antiviral treatments are available. Severe cases of RSV can be life-threatening. Ribavirin and palivizumab are the only RSV treatments approved by the US FDA (17); however, ribavirin, a broad-spectrum antiviral, has uncertain efficacy against RSV and is associated with numerous adverse effects. In contrast, palivizumab is costly, intended solely for high-risk infants to prevent RSV, and offers limited therapeutic benefits (18). TCM classifies RSV-induced pneumonia as “pneumonia and cough” due to lung heat, making SG-GC a promising candidate for intervention (19). This study investigates the antiviral mechanism of SG-GC from the perspective of metal-organic supramolecular interactions, aiming to clarify its synergistic efficacy and provide insights for RSV drug development.

## 2 Materials and methods

### 2.1 Drugs and materials

Ephedra, gypsum, bitter almond, and licorice (200812, 201138, 191108001, 210214) were sourced from Beijing Tongrentang Nanjing Pharmacy. Reference substances, including glycyrrhizic acid, ribavirin, calcium sulfate, zinc sulfate, copper sulfate, and iron sulfate (A12HS191107, 026GS165579, S05HS193206, S24260, S70004), were obtained from Shanghai Yuanye Biotechnology Co., Ltd. Magnesium sulfate (20141231) was acquired from Shanghai Lingfeng Chemical Reagent Co., Ltd. The EDTA standard solution (M10IR16124A) was also procured from Shanghai Yuanye Biotechnology Co., Ltd. Fetal bovine serum (7E721D3) was purchased from Nanjing Novezan Biotechnology Co., Ltd. Concentrated nitric acid (20200930) was obtained from Sinopharm Chemical Reagent, while the H_2_O_2_ solution (20220601) was acquired from Tianjin Oriental Guangcheng Pharmaceutical Chemical Co., Ltd. Additionally, DMEM high-glucose culture medium and PBS buffer were sourced from Nanjing Senbeiga Biotechnology Co., Ltd.

### 2.2 Instruments

CO_2_ cell culture incubator (Formascientific, USA), enzyme label spectrophotometric plate reader (Spectra Max 190, USA), ultra-clean workbench (Suzhou Purification Equipment Factory), BX20 inverted microscope (Olympus, Japan), digital display constant temperature water bath pot (Qun'an Instrument Laboratory Co., Ltd.), fluorescence quantitative PCR instrument (Shanghai Roche Diagnostic Products Co., Ltd.), small animal living Micro CT imaging system (PerkinElmer, USA), ultra-trace spectrophotometer (Thermo Fisher Scientific, USA), inductively coupled plasma mass spectrometer (Agilent, USA), microwave digestion instrument (TANK40, Shanghai Xinyi Microwave Chemical Technology Co., Ltd., Shanghai, China).

### 2.3 Cells, viruses and experimental animals

Laryngeal epidermoid carcinoma cells Hep-2 were purchased from Wuhan Shangen Biotechnology Co., Ltd., and human non-small cell lung cancer cells A549 were purchased from Hunan Fenghui Biotechnology Co., Ltd. Human RSV strain A2 was kindly provided by the Jiangsu Provincial Key Laboratory of Pediatric Respiratory Diseases (Nanjing University of Chinese Medicine). 48 female BALB/c mice (5 weeks old, 15–18 g) were adaptively reared for 1 week before the experiment. They were purchased from Shanghai Bikai Keyi Biotechnology Co., Ltd.; ethics number: 202404A065; animal license number: SCXK (Shanghai) 2023-0009.

### 2.4 Determination of RSV virulence

Hep-2 cells were seeded in a 96-well cell plate. The following day, when the cell density reached 80%–90%, the RSV viral solution was diluted tenfold in a gradient of DMEM medium containing 2% fetal calf serum. Dilutions of 10^1^, 10^2^, 10^3^, 10^4^, 10^5^, 10^6^, 10^7^, 10^8^, and 10^9^ were inoculated into the Hep-2 cells, with each dilution replicated in eight wells and a blank control group included. After inoculation, the cells were incubated at 37°C with 5% CO_2_ for 2 h. The viral solution in the 96-well plate was then aspirated, and the wells were washed twice with PBS solution. DMEM medium containing 2% fetal calf serum was added, and the plate was placed back at 37°C with 5% CO_2_ for an additional 3–4 days. After this incubation period, the plate was examined under a microscope to observe and record the number of wells exhibiting cytopathic effects (CPE) in Hep-2 cells at each dilution. The highest dilution at which no new lesions appeared was noted. Hep-2 cells infected with serially diluted viruses displayed cytopathic effects 3–4 days later. The Reed-Muench method was employed to calculate the 50% tissue culture infectious dose (TCID_50_) of RSV. Notably, syncytial formation of RSV was observed on the third day of culture. The TCID_50_ value for RSV virulence calculated using the Reed-Muench formula was 10^−8^/0.1 ml.

### 2.5 Sample preparation

#### 2.5.1 Preparation of MXSGT

Weigh 9 g of ephedra, 24 g of gypsum, 9 g of bitter almond, and 6 g of licorice, and place them in a decoction bag within a 500 ml round-bottomed flask. Add 8 times the amount of deionized water, bringing it to room temperature. Allow the mixture to stand for 30 min, then apply strong heat to boil the liquid. Reduce to a simmer and let the mixture boil gently for 30 min, concentrating it to 40 ml. Filter the liquid through gauze while it is still hot.

#### 2.5.2 Preparation of Sg-Gc co-decoction

Weigh 24 g of gypsum and 6 g of licorice, and place them in a decoction bag within a round-bottomed flask. Add deionized water in a quantity that is eight times the weight of the solids, and allow the mixture to stand at room temperature for 30 min. Following this, apply heat to the decoction. Bring the liquid to a boil, then reduce the heat to maintain a gentle simmer for 30 min, concentrating the solution to a final volume of 40 ml. While the liquid is still hot, filter it through gauze.

#### 2.5.3 Preparation of calcium sulfate-licorice co-decoction

Weigh 24 g of anhydrous calcium sulfate and 6 g of licorice, placing them in a decoction bag within a round-bottomed flask. Add 8 times the amount of deionized water. Decoct, concentrate, and filter as described above to obtain the calcium sulfate-licorice co-decoction.

#### 2.5.4 Preparation of licorice decoction

Weigh 6 g of licorice, placing it in a decoction bag within a round-bottomed flask. Add 8 times the amount of deionized water, then boil, concentrate, and filter as previously outlined to obtain the licorice single decoction.

#### 2.5.5 Preparation of gypsum decoction

Weigh 20, 24, 30, and 60 g of gypsum into separate decoction bags and place them in 4 round-bottomed flasks. Add eight times the amount of deionized water, then decoct as previously described and concentrate the solution to 40 ml. Filter the mixture while hot to obtain gypsum single decoction with crude drug concentrations of 0.5, 0.6, 0.75, and 1.5 g/ml. Preparation of Ribavirin Positive Drug Solution: Weigh 25 mg of ribavirin into 10 ml of ultrapure water to achieve a concentration of 2.5 mg/ml ribavirin solution.

The aforementioned drugs were stored at 4°C for future use.

### 2.6 The detection of calcium ion content in gypsum decoction was performed using the ethylenediamine tetraacetic acid complex titration method

Weigh 5.4 g of ammonium chloride and dissolve it in 20 ml of distilled water. Subsequently, add 35 ml of concentrated ammonia solution and dilute the mixture with distilled water to a final volume of 100 ml to prepare the NH_3_·H_2_O-NH_4_Cl buffer. Accurately pipette 0.1 ml of gypsum decoctions at varying concentrations (0.5, 0.75, and 1.5 g/ml) into 250 ml Erlenmeyer flasks. To each flask, add 10 ml of the NH_3_·H_2_O-NH_4_Cl buffer along with a suitable amount of chrome black. Introduce an appropriate quantity of T indicator and titrate with a 1 mol/L EDTA standard solution until the solution transitions from wine red to pure blue. This determination should be performed in triplicate.

Calculation formula:

$$X=(V_{E}C_{E}M_{\mathrm{Ca}})/V_{\mathrm{Sample}}$$

X is the calcium ion content (μg/ml); $V_{E}\;$ is the volume of EDTA standard solution (ml); $C_{E}\;$ is the concentration of EDTA standard solution; $\;M_{\mathrm{Ca}}$ is the relative molecular mass of calcium ions; $V_{\mathrm{Sample}}\;$ is the sample volume (ml).

### 2.7 Detection of trace metal ion content in gypsum water decoction by inductively coupled plasma mass spectrometry

Pipette precisely 0.1 ml of gypsum decoctions at varying concentrations (0.5, 0.75, and 1.5 g/ml of crude drug) into microwave digestion tanks. Add 0.8 ml of concentrated nitric acid and 0.1 ml of H_2_O_2_ solution, then place the tanks in the microwave digestion instrument. Follow the digestion procedure as outlined in Supplementary Table S1. After digestion, adjust the volume to 10 ml in a volumetric flask, shake well to prepare the test solution, and measure the concentrations of the four metal ions (Mg, Al, Fe, and Zn) using ICP-MS, ensuring that each measurement is conducted in triplicate.

### 2.8 Grouping of mice, modeling and drug administration

Feeding of mice: The temperature is maintained at 25 ± 5°C, the relative humidity is kept at 60 ± 5%, and the light cycle is set to 12 h. Mice are provided with mouse-specific feed and have free access to food and water.

Mice grouping and modeling: A total of 48 five-week-old BALB/c mice were acclimatized for one week and subsequently randomized into eight groups: control group (Control), model group (Model), gypsum group (SG), licorice group (GC), SG-GC group (SG-GC), calcium sulfate-licorice group (CaSO_4_-GC), MXSGT group (MXSGT), and positive drug group (Ribavirin), with six animals in each group. The mice were anesthetized using isoflurane gas. Mice in the model and administration groups were intranasally infected with 80 μl of RSV virus at a titer of 10^6^ TCID_50_ per mouse, while those in the normal group received 80 μl of normal saline via the same route.

Administration to mice: mice in each group were administered intragastrically at 2 h post-modeling, and this treatment was continued for 3 consecutive days. The same volume of normal saline was administered to both the blank and model groups. Dosages were calculated based on the conversion ratio of adult (60 kg) drug dosage to the body surface area of mice (20 g). The dosages for each group were consistent with that of the MXSGT group. The body weight of mice in each group was recorded daily.

The dosages assigned to the mice in each experimental group were as follows: mice in the Ribavirin group received a dosage of 46 mg/kg, a value corroborated by the data from reference. The MXSGT-treated mice were administered a dosage of 18 g/kg, as previously reported in reference. In both the SG-GC and CaSO_4_-GC groups, the dosages were set at 11 g/kg each. The SG group received a dosage of 9 g/kg, while the GC group was subjected to a lower dosage of 2.25 g/kg. These precisely determined dosages are critical for comprehensively evaluating the specific impacts of each treatment regimen on the experimental outcomes.

### 2.9 Micro CT scanning of mouse lungs

According to previous studies on Micro CT scanning of mouse lungs (20), the following procedures were carried out: on the second day, eight groups of mice underwent CT imaging experiments, utilizing high-resolution x-ray imaging technology. Prior to scanning, isoflurane is administered to anesthetize the mouse. The mouse is then swiftly positioned on the scanning bed, and a mask filled with isoflurane gas is placed over its face. The scanning position is fine-tuned using a micro-adjuster to ensure that the lungs are oriented at the optimal angle for a 360° scan.

### 2.10 Mouse material collection

The mice were fasted and deprived of water following the last administration. On the third day of modeling, six mice from each group were euthanized by dislocation. After blunt separation of the skin, fresh lung tissue was photographed and examined, and the right lungs of the mice were harvested. The middle lobe was utilized for H&E section staining to assess lung pathology, while the remaining lung tissue was frozen at −80°C for future analysis.

### 2.11 Pathological examination of mouse lung tissue

The middle lobe of the right lung from each group of mice was placed in 1 ml of 4% paraformaldehyde within a centrifuge tube, fixed at 4°C for 24 h, dehydrated, embedded in paraffin, and sectioned onto a glass slide. During hematoxylin and eosin (H&E) staining, hematoxylin stains the cell nuclei blue, while eosin stains the cytoplasm and connective tissue a light pink, facilitating the observation of pathological conditions, which were subsequently examined and photographed using a microscope.

### 2.12 Real-time quantitative PCR method to detect viral load

#### 2.12.1 Sample pretreatment and extraction of RNA

Weigh 50 mg of mouse lung tissue into a sterile, enzyme-free 1.5 ml centrifuge tube. Add 500 μl of FreeZol Reagent lysis solution and homogenize using a ball mill for 10 min. Allow the mixture to stand at room temperature for an additional 10 min. Next, add 100 μl of dilution buffer solution, shake to mix, and centrifuge at 11,200 rpm for 15 min. Transfer 400 μl of the supernatant into a new sterile, enzyme-free centrifuge tube. Add an equal volume of isopropyl alcohol, mix by inverting, and let it stand at room temperature for 10 min before centrifuging at 11,200 rpm for 10 min. Carefully discard the supernatant. Wash the precipitate by adding 1 ml of 75% ethanol, followed by centrifugation at 9,100 rpm for 3 min at room temperature. Discard the supernatant, repeat the washing process twice, and subsequently discard the supernatant. Allow the precipitate to dry at room temperature, then add 20 μl of enzyme-free water to dissolve the precipitate, vortexing for 3 min to ensure complete dissolution. Store the solution at −80°C.

#### 2.12.2 Determination of RNA concentration

Take 1 µl of the total RNA sample from each tube and measure the RNA concentration and the A_260_/A_280_ ratio using an ultra-micro-volume spectrophotometer. A ratio in the range of 1.8–2.0 indicates that the RNA is of sufficient quality. Use enzyme-free water to dilute each sample to a total RNA concentration of 200 ng/μl.

#### 2.12.3 Reverse transcription of RNA into cDNA

Prepare the following mixture in a sterile, enzyme-free PCR tube: ×μl RNA (1 μg), 4 μl of 4 × gDNA Wiper Mix, and (16-x) μl of RNase-free ddH_2_O. Gently mix by pipetting and heat at 42°C for 2 min. Directly add 4 μl of 5× Hiscript II qRT SuperMix II to the reaction tube from the previous step, and gently mix with a pipette. Heat at 50°C for 15 min, then at 85°C for 5 s, and finally hold at 4°C. store at −20°C for long-term use.

qPCR detection: quantitative PCR was performed using GAPDH as the internal reference. The primer sequences utilized are detailed in Supplementary Table S2. The reaction conditions utilized are detailed in Supplementary Table S3. Prepare 20 µl reaction system in a qPCR tube. The quantified viral RNA levels were presented as a percentage relative to the control.

### 2.13 Detects the effect of drugs on cell survival rate

Sample preparation: Dissolve 13.2 mg of GA in 10 ml of DMEM high-sugar medium to prepare a 1,600 μM GA solution. Pass the solution through a 0.22 μm sterile filter to eliminate impurities and ensure sterilization. Subsequently, dilute the solution with DMEM to achieve final concentrations of 25, 50, 100, 200, 400, 600, and 800 μM. Weigh and dissolve standards of calcium sulfate, zinc sulfate, copper sulfate, iron sulfate, and magnesium sulfate in DMEM, diluting each to a concentration of 50 μM. Additionally, dilute gypsum decoctions of 500, 750, and 1,500 mg/ml with DMEM to final concentrations of 250, 375, and 750 mg/ml, respectively.

CCK-8 detection: Add 100 µl of A549 cell suspension at a density of 2 × 10^4^ cells/ml to each well of a 96-well plate. Pre-incubate the culture plate in an incubator for 24 h at 37°C with 5% CO_2_. After removing the culture plate from the culture bottle, discard the cell supernatant from each well and wash the wells with PBS solution. Next, inoculate 200 µl of the diluted sample into each well of the 96-well plate, ensuring that there are 6 replicate wells for each concentration. Additionally, set up control wells for the cells. Once all wells are prepared, label them on the 96-well plate and place the plate in a 37°C incubator with 5% CO_2_ for culture. After 24 h, terminate the culture by removing the 96-well plate from the incubator, discarding the supernatant from each well, and washing the wells twice with PBS solution. Prepare a mixture of CCK-8 and medium in the appropriate proportion, then add 100 µl of this mixture to each well. Incubate the culture plate in the incubator for 1 h. Finally, use a microplate reader to measure the absorbance at 450 nm.

Calculation formula:

$$\mathrm{cell}\;\mathrm{survival}\;\mathrm{rate}=\frac{A_{s}\text{-}A_{b}}{A_{c}\text{-}A_{b}}$$

$$\begin{array}{rl} A_{s} & :\mathrm{Absorbance}\;\mathrm{of}\;\mathrm{test}\;\mathrm{wells}\;(\mathrm{including}\;\mathrm{cells},\;\mathrm{culture}\;\mathrm{medium},\;\mathrm{CCK}\\ & \;\text{-}8\;\mathrm{solution}\;\mathrm{and}\;\mathrm{drug}\;\mathrm{solution}) \end{array}$$

$$\begin{array}{rl} A_{c} & :\mathrm{Absorbance}\;\mathrm{of}\;\mathrm{control}\;\mathrm{wells}\;(\mathrm{including}\;\mathrm{cells},\;\mathrm{culture}\;\mathrm{medium},\;\mathrm{CCK}\text{-}8\;\\ & \;\mathrm{solution},\;\mathrm{excluding}\;\mathrm{drugs}) \end{array}$$

$$\begin{array}{rl} A_{b} & :\mathrm{Absorbance}\;\mathrm{of}\;\mathrm{blank}\;\mathrm{wells}\;(\mathrm{containing}\;\mathrm{culture}\;\mathrm{medium},\;\mathrm{CCK}\text{-}8\;\\ & \;\mathrm{solution},\;\mathrm{excluding}\;\mathrm{cells}\;\mathrm{and}\;\mathrm{drugs}) \end{array}$$

### 2.14 *In vitro* anti-RSV experiment

To determine the sub-inhibitory concentration of GA, we pre-conducted an antiviral preliminary experiment with GA acting alone: the concentration gradient of GA was set at 10, 40, 80, 100, and 200 μM, and its inhibition rate against RSV was detected.

Add 0.8 ml of A549 cell suspension, with a density of 10^5^ cells/ml, to each well of a 24-well plate. Incubate the plate in a 37°C, 5% CO_2_ environment for 24 h. Prepare the gypsum decoction by diluting it with DMEM containing 2% fetal calf serum to achieve crude drug concentrations of 0.25, 0.375, and 0.75 g/ml. Additionally, prepare GA solutions at varying concentrations (10, 40, 80, 100, and 200 μM), along with 50 μM solutions of calcium sulfate, zinc sulfate, copper sulfate, iron sulfate, and magnesium sulfate for administration. Once the cells have formed a monolayer, add each drug and incubate the cells for 1 h. Concurrently, incubate 100 TCID_50_ of RSV virus along with the drugs for 1 h. After this incubation, aspirate the liquid and wash the cells with PBS. Subsequently, add the RSV virus solution to infect the cells for 2 h. Following this, aspirate the virus solution, wash the cells three times with PBS to remove any unabsorbed virus, and then add the drugs to the cells. Incubate the cells for an additional 24 h in a 37°C, 5% CO_2_ incubator. Finally, lyse the cells using lysis buffer and detect the viral load via qRT-PCR, following the same methodology as described above.

## 3 Result

### 3.1 Evaluation of the anti-RSV efficacy of SG-GC *in vivo*

#### 3.1.1 Detection of viral load

**Figure 1:**
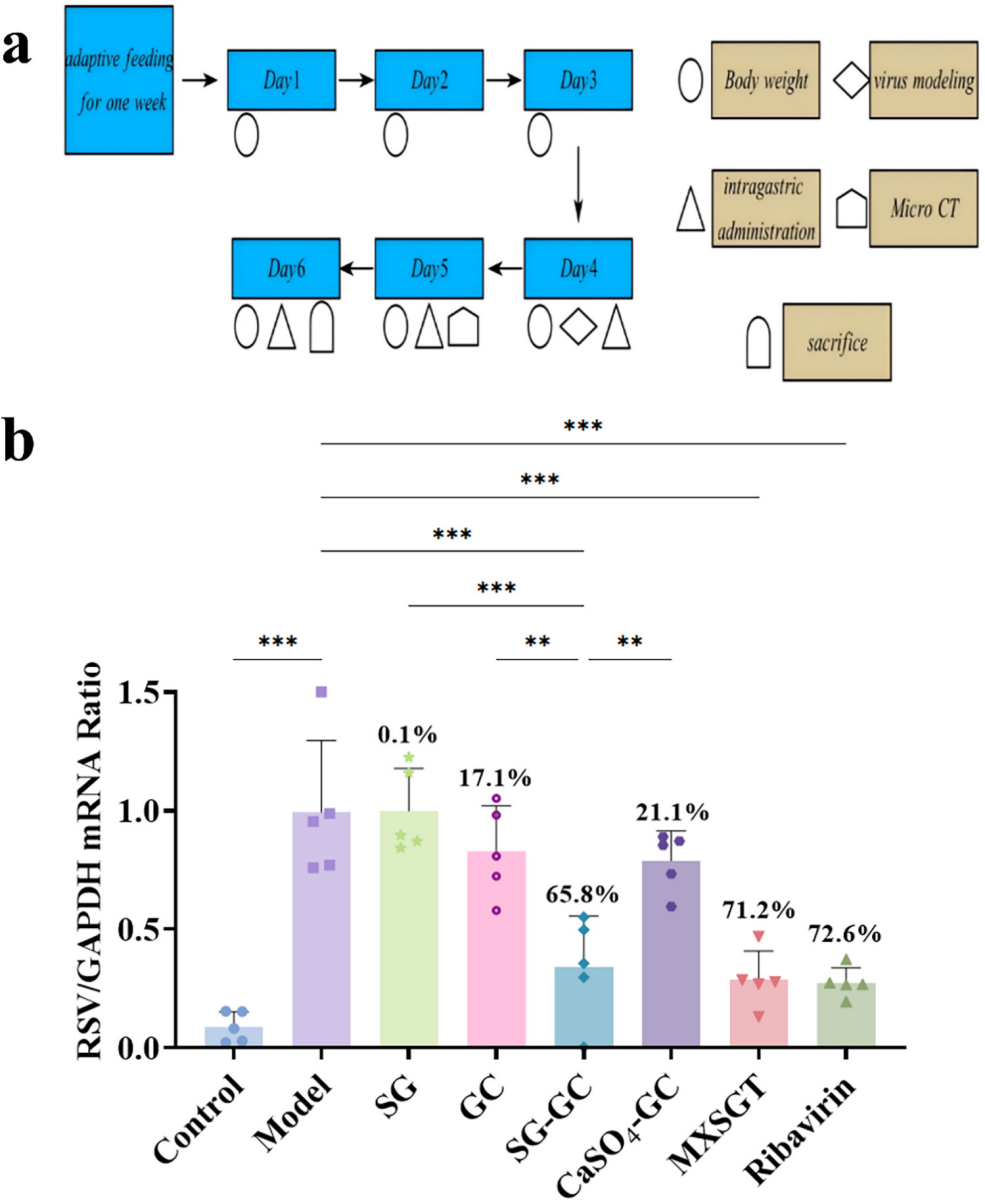
Study on the *in vivo* anti-RSV effect of drugs in each group. **(a)** Schematic diagram of mouse modeling and drug administration process. **(b)** RSV virus load in lung of mice in each group (*n* = 5). * *p* < 0.05, ** *p* < 0.01, *** *p* < 0.001.

Perform relevant operations through specific methods, as shown in Figure 1A. The RT-qPCR method was employed to quantify the viral load in lungs of mice across all experimental groups. As illustrated in Figure 1B, the viral inhibition rates for the positive control drugs Ribavirin, MXSGT, and the SG-GC pair were 72.6%, 71.2%, and 65.8%, respectively, all demonstrating significant inhibitory effects (*p* < 0.001). In contrast, the inhibition rates for single-flavored gypsum and single-flavored licorice were not significant. Notably, the antiviral effect of the SG-GC group was markedly superior to that of the SG group (*p* < 0.001) and the GC group (*p* < 0.01), highlighting the characteristics of compatibility and synergy. Given that the inhibition rate of the calcium sulfate-licorice group was only 33%, it is preliminarily suggested that the enhanced inhibition rate observed in the SG-GC group (*p* *<* 0.05) may be attributed to the trace elements present in gypsum.

#### 3.1.2 Observation of lung damage

**Figure 2:**
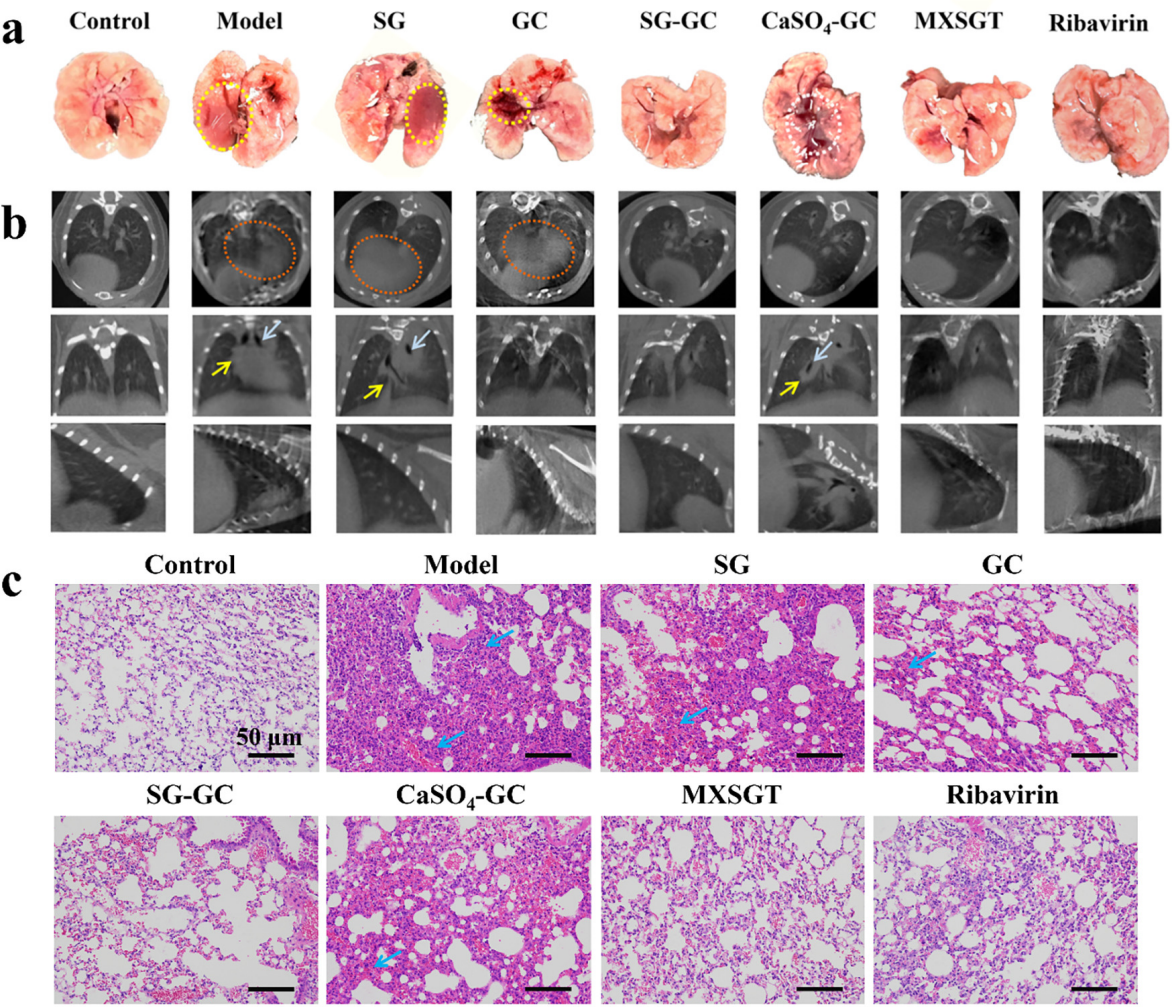
Observation of lung injury of mice in each group. **(a)** Fresh lung tissue morphology. **(b)** Lung micro-CT scanning image. **(c)** Pathological changes of lung tissue in mice in each group.

Lung damage was assessed through examination of fresh lung tissue. Figure 2A illustrates that the fresh lung tissue from the model group, SG group, and GC group exhibited dark red congestion and edema that were visible to the naked eye (indicated by the yellow circle). In contrast, the fresh lung tissue of the CaSO_4_-GC group displayed dark red lesions at the junction of the lung lobes (marked by the white circle), although the extent and severity of damage were reduced compared to the model group. As illustrated in Figure 2B, CT scans revealed a “white lung” phenomenon (highlighted by the red circle), lung consolidation (indicated by the yellow arrow), and bronchiectasis (shown by the white arrow), characterized by flocculent and ground-glass opacities diffusely distributed throughout both lungs. The lung tissue in the ribavirin group, MXSGT group, and SG-GC group appeared normal and was light pink in color, with no significant pulmonary capillary congestion, edema, or hemorrhage observed. The lung tissue damage was minimal, and no notable pathological findings were evident on CT scans. These results indicate that mice treated with the SG-GC drug pair not only reduce viral load but also exhibit effects comparable to those of the full prescription in alleviating symptoms of lung infection.

#### 3.1.3 Pathological changes in lung tissue

The H&E staining results of mouse lung tissue sections from each group are presented in Figure 2C. The model group, SG group, GC group and CaSO_4_-GC group exhibited alveolar wall rupture and congestion, accompanied by significant inflammatory cell infiltration between the alveoli (indicated by the blue arrow). After treatment, the ribavirin group, MXSGT group, and SG-GC group showed pathological conditions of the lung tissue that were comparable to those of the blank group. This suggests a marked improvement in inflammatory cell infiltration, with the alveolar wall structure remaining largely intact. These findings indicate that the SG-GC drug treatment not only reduced viral load but also exhibited effects similar to those of the full prescription in alleviating symptoms of pulmonary infection. In contrast, the CaSO_4_-GC group did not demonstrate these characteristics. Therefore, it is speculated that Ca^2+^ is not the primary active substance of the SG-GC drug against RSV, and the observed effects may be related to trace metal elements.

#### 3.1.4 Changes in mouse body weight

**Figure 3:**
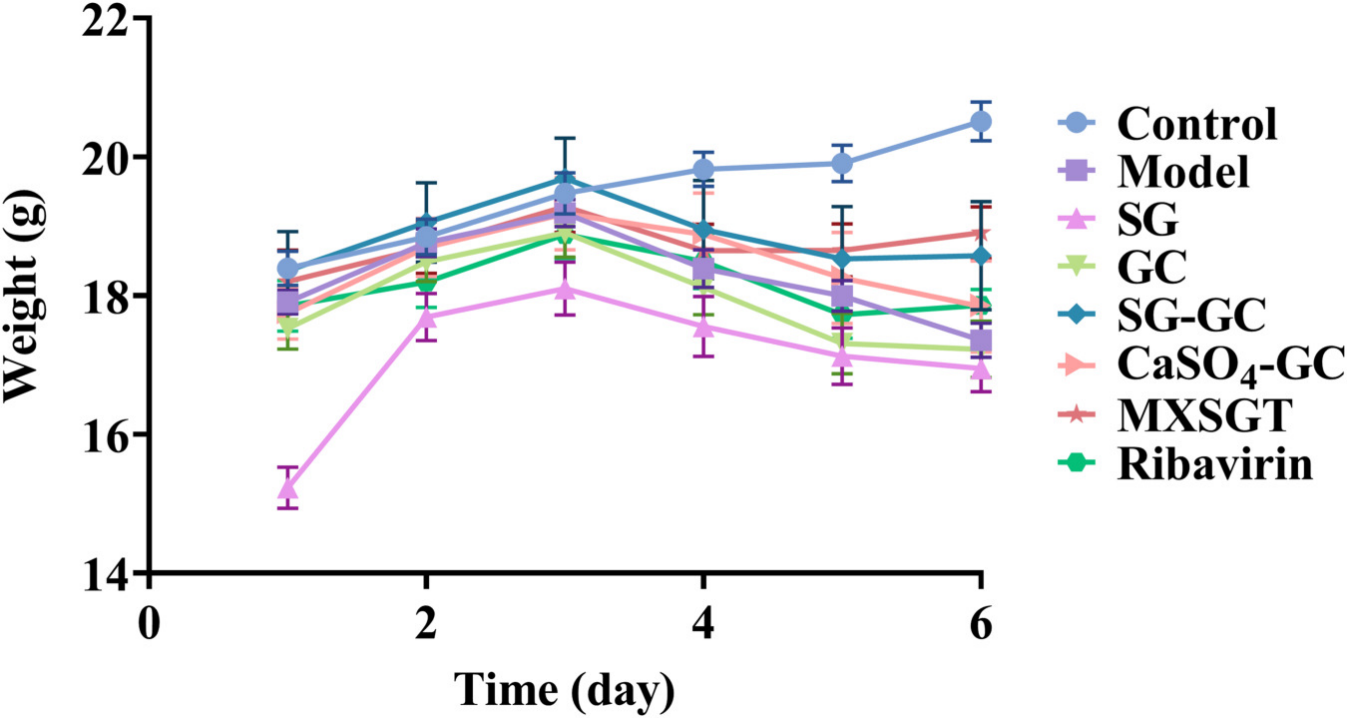
Body weight of mice in each group ($\bar{x}\pm s$, *n* = 6).

Mice may experience a decreased appetite and subsequent weight loss during viral infections. As illustrated in Figure 3, the mice in the blank (Control) group exhibited a gradual increase in weight. After modeling on day 3, mice in all other groups showed a weight decrease. Notably, by day 6, the body weight of treated mice in the ribavirin, MXSGT, and SG-GC groups, which have antiviral efficacy, showed a slight increase.

### 3.2 Evaluation of the *in vitro* anti-RSV efficacy of Sg-Gc on the main component

#### 3.2.1 Effect of GA on the anti-RSV efficacy of gypsum

**Figure 4:**
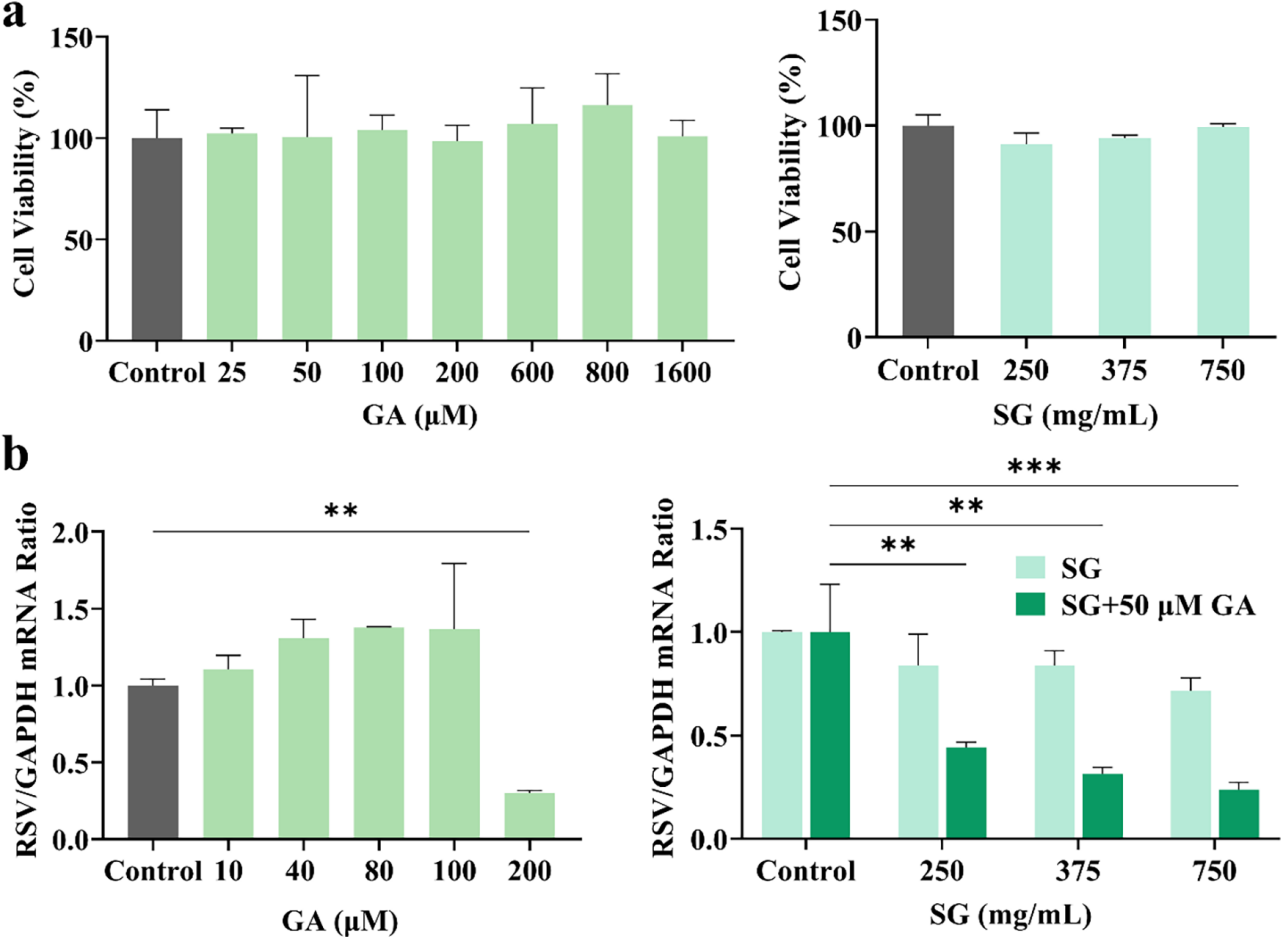
*In vitro* antiviral effect of GA and gypsum decoction **(a)** on cell viability **(b)** on RSV viral load. * *p* < 0.05, ** *p* < 0.01, *** *p* < 0.001.

GA, the principal component of licorice, interacts with various metal ions present in the gypsum decoction. This interaction leads to the formation of diverse metal-organic supramolecular complexes, which may alter pharmacological activity. Initially, the cytotoxic concentration range of each substance on host cells was assessed using CCK-8 assays. As illustrated in Figure 4A, GA exhibited no cytotoxicity within the concentration range of 25–1,600 μM, while SG demonstrated similar safety between 250 and 750 mg/ml. Subsequently, the viral load in the cells was measured through RT-qPCR experiments. As depicted in Figure 4B, when the concentration of GA was 100 μM or lower, its inhibition rate against RSV remained below 20%, indicating that GA alone exerted no significant antiviral activity within this range. Only at concentrations of 200 μM or higher did the inhibition rate increase to 75%. Based on these findings, 50 μM was chosen as the GA concentration for combination experiments. This concentration, while lacking significant standalone antiviral effects, is capable of demonstrating synergistic potential through interactions with SG. SG exhibited some anti-RSV activity, although this effect was not statistically significant, with inhibition rates for RSV decoctions containing varying amounts of crude drug remaining below 25%.

However, the anti-RSV effect was significantly enhanced when GA was combined with SG at a sub-inhibitory concentration of 50 μM. At SG crude drug concentrations of 0.25 g/ml, 0.375 g/ml, and 0.75 g/ml, the inhibition rates increased to 56%, 69%, and 77%, respectively, which is more than double that observed at the same concentration, indicating a certain concentration dependence. This phenomenon is analogous to the synergistic properties of the SG-GC drug pair. Importantly, this synergy does not appear to be directly attributed to GA; rather, it may result from the interaction of the metal ion complex with the gypsum decoction.

#### 3.2.2 The effect of GA on the anti-RSV efficacy of various metal ions in gypsum

**Figure 5:**
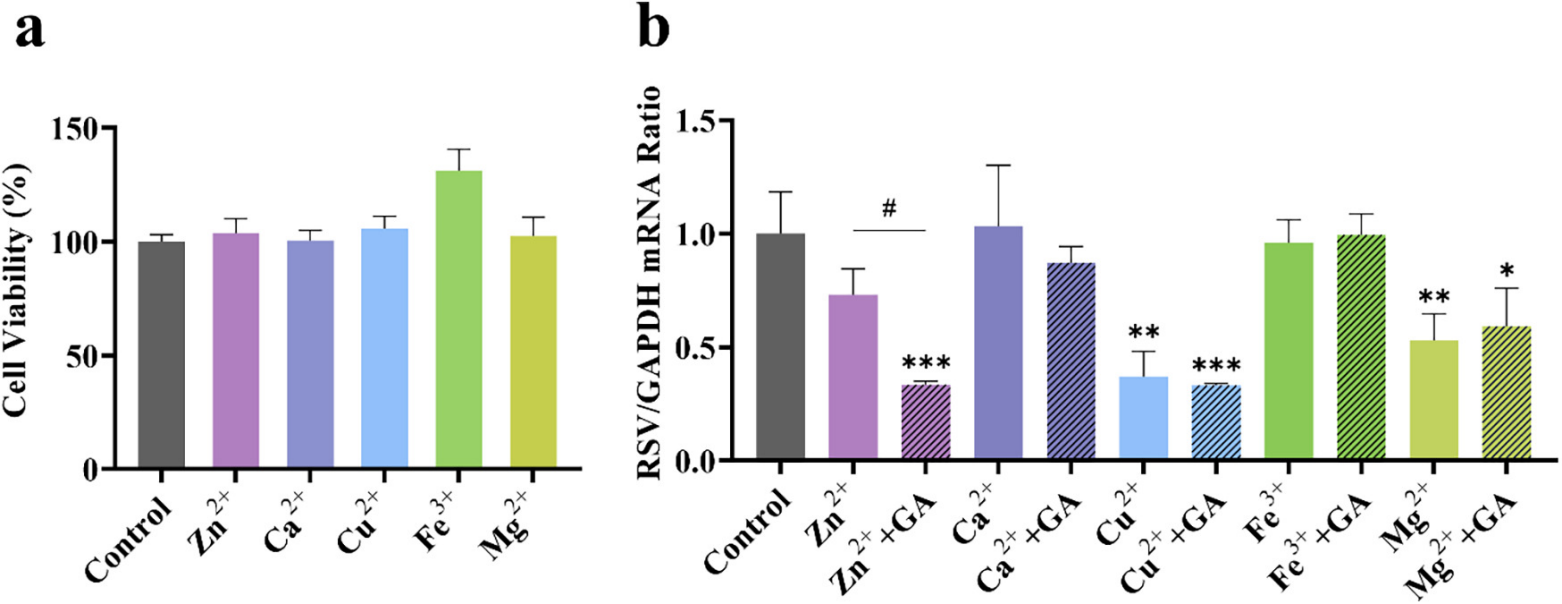
Effect of different metal ions (50 μM) **(a)** on cell viability and **(b)** on RSV viral load in the presence of 50 μM GA. *p < 0.05, **p < 0.01, ***p < 0.001.

To further investigate the compatibility and synergistic effects of metal ions present in the gypsum decoction with GA, this study focuses on the key role of metal-organic supramolecules formed by metal ions and GA. Accordingly, we selected Ca^2+^, Cu^2+^, Mg^2+^, Fe^3+^, and Zn^2+^ as representative research objects to systematically examine their anti-RSV activity. As illustrated in Figure 5A, at a metal ion concentration of 50 μM, host cell safety was satisfactory; however, the anti-RSV effects varied (Figure 5B). Ca^2+^ and Fe^3+^ exhibited negligible direct anti-RSV activity, while Cu^2+^ and Mg^2+^ demonstrated inhibition rates of 63% and 47%, respectively. This suggests that these ions possess some inherent anti-RSV properties, but their effectiveness remains largely unchanged when combined with sub-inhibitory concentrations of GA (50 μM). Notably, the inhibition rate of 50 μM Zn^2+^ against RSV was 27%, which increased to 66% when combined with 50 μM GA, representing a 2.4-fold enhancement compared to the inhibition rate observed with Zn^2+^ alone. These findings indicate that the gypsum decoction itself exhibits a certain antiviral effect, likely attributable to trace metal elements such as copper, magnesium, and zinc. However, only the combination of Zn^2+^ and GA displayed synergistic properties akin to those of the SG-GC pair, suggesting that the Zn-GA supermolecule formed through the coordination of Zn^2+^ and GA may be a critical factor in the antiviral mechanism of the SG-GC pair. Notably, other trace metal ions in gypsum (such as Mn^2+^, Al^3+^, etc.), though not systematically investigated in this study, may still hold potential as antiviral components based on their inherent biological activity, providing new directions for future research.

#### 3.2.3 Determination of metal element content in gypsum water decoction with varying crude drug concentrations

**Figure 6:**
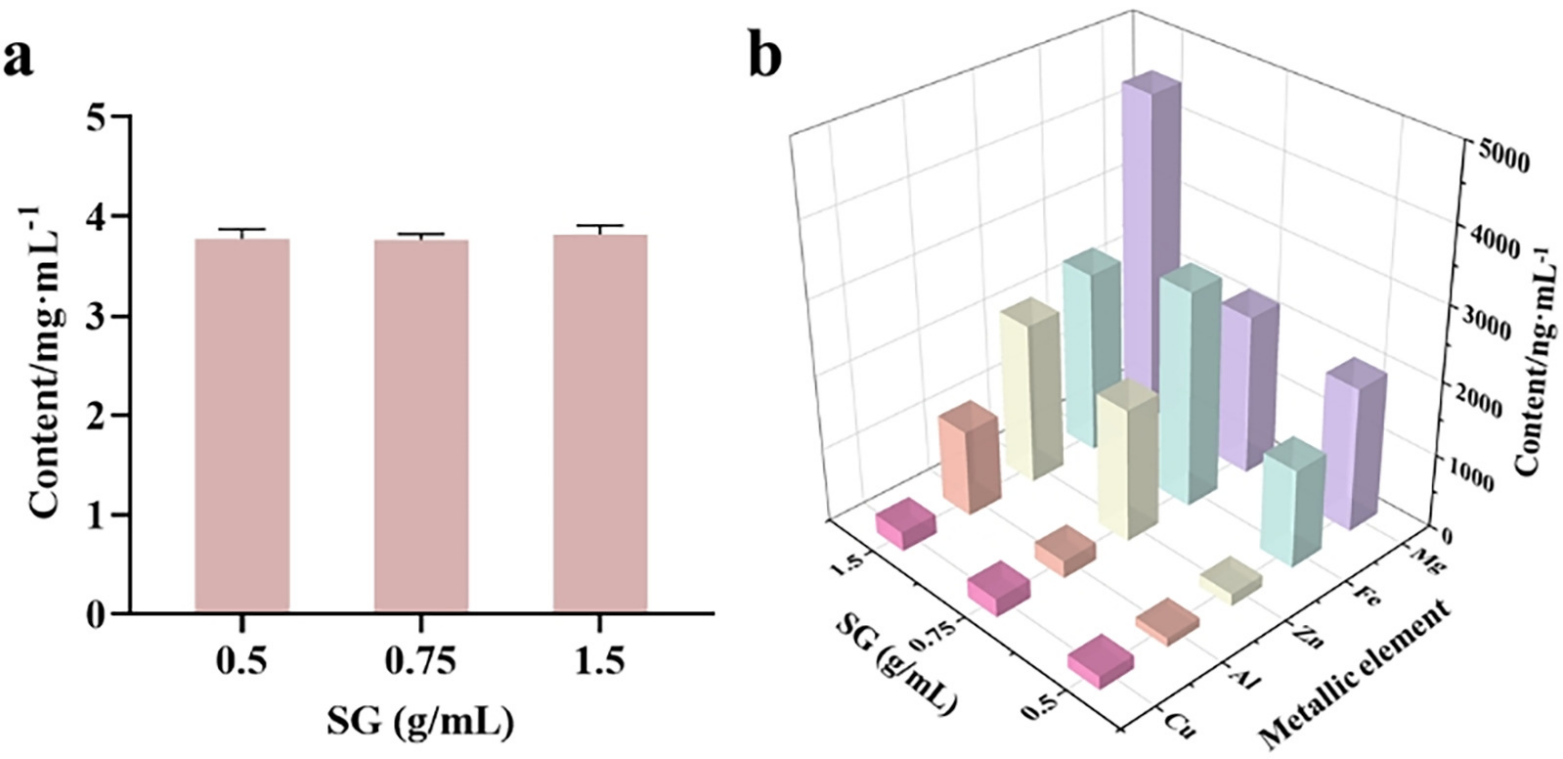
Determination of metal ion concentration in gypsum decoction. **(a)** Determination of Ca^2+^ in gypsum decoction by EDTA complexation titration; **(b)** Determination of Cu, Mg, Al, Fe and Zn in gypsum decoction by ICP-MS.

The determination of metal element content in gypsum water decoction involves titrating the Ca^2+^ concentration with an EDTA standard solution, while trace elements are quantified using ICP-MS. Results presented in Figure 6A indicate that as the amount of gypsum increases (simulating the reuse of gypsum in the recipe), the Ca^2+^ concentration in the decoction remains relatively constant at 3.8 g/L, suggesting that the CaSO_4_ in the decoction has reached saturation. Additionally, Figure 6B demonstrates that with an increase in gypsum quantity, the concentrations of trace elements Cu and Fe in the decoction exhibit a slight increase, whereas the levels of Mg, Al, and Zn increase significantly. Specifically, when the amount of gypsum increased threefold, the concentration of Cu^2+^ rose from 183 ng/ml to 262 ng/ml, representing an increase of 1.4 times. Similarly, the concentration of Fe^3+^ increased from 1,335 ng/ml to 2,467 ng/ml, reflecting an increase of 1.8 times. The Mg^2+^ concentration showed a notable increase from 1960 ng/ml to 4,480 ng/ml, a rise of 2.3 times. The concentration of Al^3+^ increased significantly from 120 ng/ml to 1,172 ng/ml, which is an increase of 9.7 times. Particularly noteworthy is the surge in Zn^2+^ concentration, which escalated from 166 ng/ml to 2,180 ng/ml, indicating a remarkable increase of 13.1 times, the highest among the trace elements. This observation suggests that as the amount of gypsum increases, the content of trace elements generally rises. Consequently, the anti-RSV effect of varying amounts of SG combined with GA is dependent on concentration (Figure 4B). Furthermore, while the trace elements may contribute to gypsum's anti-RSV efficacy, its compatibility with licorice is also a crucial factor in enhancing this efficacy.

## 4 Discussion

This study focuses on the sovereign-courier medicinal pair SG-GC within MXSGT as its primary research subject. Using a mouse model of RSV infection, the findings demonstrate that both MXSGT and the SG-GC drug pair exhibit significant anti-RSV effects. Specifically, both treatments resulted in reduced viral loads in the lungs of mice, alleviated lung injury symptoms, and decreased inflammatory cell infiltration. In contrast, the antiviral effects of the individual components, gypsum or licorice, were found to be minimal, suggesting that gypsum must be used in conjunction with licorice to effectively exert its antiviral properties. A comparative analysis between the SG-GC medicinal pair and the CaSO_4_-GC group indicated that the antiviral efficacy may derive from trace metal elements present in gypsum. To further investigate the compatibility principle of gypsum and licorice, an RSV-infected cell model was utilized to examine the interactions between mineral and organic components. After confirming the safety of each component on host cells, it was observed that gypsum alone exhibited limited inhibitory potential against RSV, with an inhibition rate ranging from 16.2% to 28.4%. However, when combined with GA at a sub-inhibitory concentration (50 μM), the anti-RSV effect was significantly enhanced, improving by 2.7–4.3 times. Subsequent studies revealed that the inhibitory effects of metal ions (Ca^2+^, Fe^3+^, Cu^2+^, Mg^2+^, Zn^2+^) in the gypsum decoction varied. At equivalent concentrations, Cu^2+^ demonstrated the most effective inhibition, although no enhancement was noted when combined with GA. In contrast, the combination of Zn^2+^ with GA resulted in an increased inhibitory effect of up to 2.4 times, showcasing a compatibility enhancement similar to that observed with the SG-GC drug pair. In addition, ICP-MS analysis confirmed that as the amount of gypsum increases, the concentration of trace elements also rises, with Zn^2+^ exhibiting the most pronounced increase. This finding suggests, from another perspective, that one potential purpose of reusing gypsum may be to enhance the content of trace elements. While Zn^2+^ has been extensively documented for its antiviral properties, this study is the first to reveal a significantly enhanced antiviral effect when it is combined with GA.

**Figure 7:**
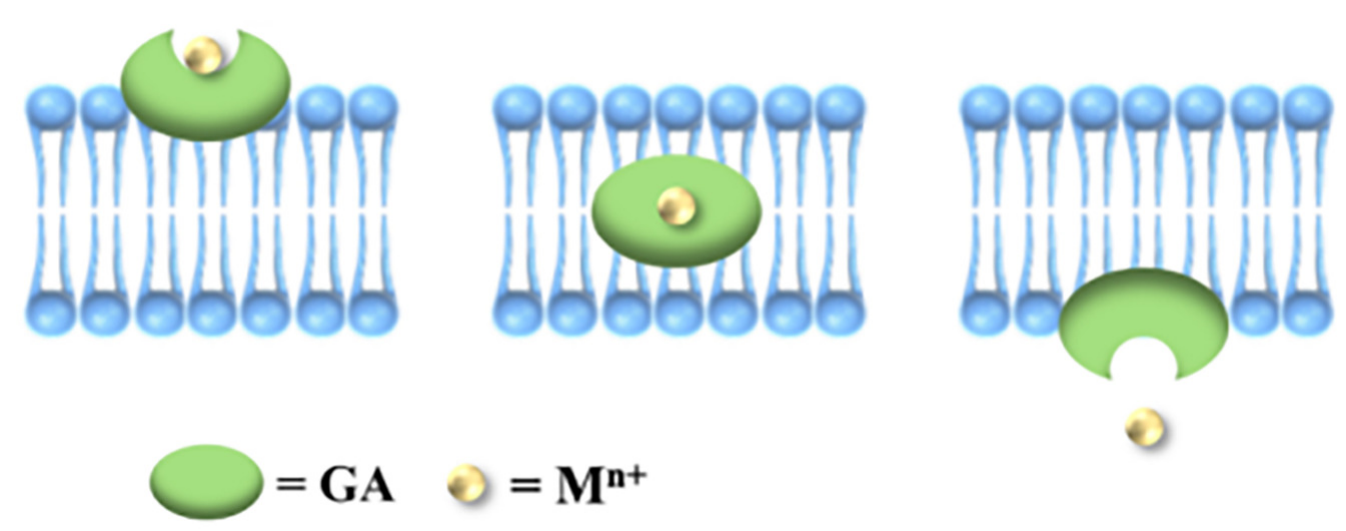
Schematic diagram of ionophore assisting metal ions to enter cells.

From the perspective of metal-organic supramolecular chemistry, GA contains carboxyl, hydroxyl, and other functional groups that can coordinate with metal ions. However, the spatial arrangement of the three carboxyl groups in GA is such that they are too distant from one another to form a multiradical ligand group, resulting in lower stability compared to cyclic structures formed by chelating agents like ethylenediamine and ethylenediaminetetraacetic acid (EDTA). Consequently, dissociation is more likely to occur. Conversely, this characteristic also facilitates the release of metal ions from the GA complex. GA molecules possess a hydrophobic triterpene skeleton and hydrophilic sugar groups, resulting in an amphiphilic structure that exhibits effective cell membrane penetration. The hydrophobic component can integrate into the phospholipid bilayer of the cell membrane, disrupting its structure and thereby enhancing drug permeability. This study posits that GA molecules may facilitate the translocation of Zn^2+^ across the cell membrane, subsequently releasing Zn^2+^ within the intracellular environment. This function resembles that of an ionophore, as illustrated in Figure 7. Metal-organic supramolecules can be classified into chelating agents (such as EDTA), metal shuttles (like porphyrin derivatives), and ionophores based on the type of ligands (21, 22), as detailed in Table 1. Notably, only ionophores are capable of repeatedly transporting metal ions across the membrane, thereby enhancing the intracellular concentration of these ions. In this study, it was observed that the anti-RSV efficacy was augmented when GA was combined with Zn^2+^, potentially linked to the ionophore-like properties of GA. However, whether GA functions as a specific zinc ionophore warrants further investigation. Notably, we conducted supplementary studies using membrane transport experiments (23). We found that GA can act on the cell membrane and promote the transmembrane transport of Zn^2+^, which is consistent with the characteristics of ionophores.

**Table 1:** Classification of metal-organic complexes according to ligand type.

Type of ligand	Function of ligand	Physiological activity of metal ions
Metal chelator	Non-selective reduction of metal ion concentration inside and outside the cell	Reduced drug efficacy
Metal shuttle	May help extracellular metal ions cross the membrane, but cannot release free metal ions	No change
Metal Ionophores	May help extracellular metal ions cross the membrane and release free metal ions after entering the cell	Enhanced drug efficacy

Gypsum, a mineral medicine, serves as a rich source of metal ions. When utilized in medicinal applications, gypsum readily forms various metal-organic supramolecular complexes with herbal medicines. Notably, the metal-GA supramolecular complex generated from the combination of gypsum and licorice has been the subject of prior studies (24) However, existing research has yet to investigate the synergistic, antagonistic, or novel bioactive properties that may arise from the formation of metal-organic supramolecular structures due to the interaction between metal ions and organic components in gypsum. This study focuses on the supramolecular assembly phenomenon previously observed in research concerning the antiviral efficacy of MXSGT. It represents the first exploration of the antiviral compatibility mechanism of the SG-GC drug pair through the lens of metal-organic supramolecular substances, aiming to elucidate the antiviral compatibility mechanism of this pairing. The supramolecular interactions between trace metal ions and licorice, particularly the coordination and transmembrane transport of Zn^2+^ and GA, are pivotal for achieving compatibility and synergy. Furthermore, this study posits that research on the material basis of gypsum should not be conducted in isolation, but rather in conjunction with its compatible medicinal pairs or prescriptions. The metal-organic supramolecular theory marks a significant advancement in compatibility research. It should be noted, however, that systematic data on the pharmacokinetic characteristics of the SG-GC combination in humans, such as absorption rate, tissue distribution, metabolic pathways and excretion patterns, are still lacking. Its potential toxicity, especially the risk of metal ion accumulation during long-term use, remains unclear. This is a gap that urgently needs to be filled in clinical translation. Nevertheless, based on the traditional experience of TCM compatibility and preliminary animal experiments, it is hypothesized that the mild and moderating property of licorice may reduce the irritancy of metal ions in gypsum (25), embodying the TCM concept of “reducing toxicity through compatibility” and providing a direction for subsequent safety research.

Although this study found that SG-GC and MXSGT exhibit certain equivalence in direct antiviral efficacy, this does not imply that ephedra and bitter almond can be entirely excluded from the prescription. These medicinal components may play a significant role in alleviating coughs, asthma, and enhancing symptoms associated with viral infections. The precise compatibility of the four medicinal materials contributes to the effectiveness of the antiviral prescription MXSGT. Classic TCM prescriptions possess both enduring traditional advantages and unique clinical experiences in managing antiviral infections. In contemporary times, there is a need for scientific interpretations of their compatibility theories and pharmacodynamic mechanisms to facilitate the advancement of these ancient classic prescriptions. This study, based on the unique perspective of “organic-metal supermolecules”, offers a preliminary scientific explanation of the compatibility theory underlying MXSGT in TCM. This study shares common ground with existing research on metal-organic supramolecular antiviral agents. For example, Kreiser et al. found that polyphenol-zinc complexes can inhibit the replication of respiratory RNA viruses (14), both demonstrating the antiviral potential of metal-organic supramolecules. However, the core difference between the two lies in the research system: Kreiser et al. focused on exogenously chemically synthesized supramolecular complexes, whereas this study focuses on endogenously formed supramolecules in the traditional Chinese medicine compatibility system. This is highly consistent with the TCM theory of “synergistic effect through compatibility” and explores how natural medicines optimize their efficacy through supramolecular interactions in traditional compatibility. It is anticipated that this will inspire new ideas for fundamental research in TCM and provide novel antiviral strategies. The application of innovative TCM opens up new possibilities.

## Data Availability

The raw data supporting the conclusions of this article will be made available by the authors, without undue reservation.
