# Supplementary material for: Exploration of the antiviral mechanism of gypsum-licorice compatibility pairing in Ma-Xing-Shi-Gan decoction from the perspective of metal-organic supramolecular interactions
Source: Front Med Technol. 2025 Sep 1;7:1651390. doi: 10.3389/fmedt.2025.1651390 (PMC12434118; doi:10.3389/fmedt.2025.1651390)
Supplement: Supplementary file 1 [file Table1.docx]

Supplementary Material

Exploration of the Antiviral Mechanism of Gypsum-Licorice Compatibility Pairing in Ma-Xing-Shi-Gan Decoction from the Perspective of Metal-Organic Supramolecular Interactions

**Table S1** Microwave digestion procedure

| **Step** | **time（min）** | **temperature（℃）** | **stress（atm）** |
| --- | --- | --- | --- |
| **1** | 5 | 120 | 5 |
| **2** | 10 | 150 | 10 |
| **3** | 12 | 200 | 20 |
| **4** | 30 | 220 | 30 |

**Table S2** qPCR primer sequence

| **Primer names** | **primer sequences（5’→3’）** |
| --- | --- |
| GAPDH-F | AGGTCGGTGTGAACGGATTTG |
| GAPDH-R | TGTAGACCATGTAGTTGAGGTCA |
| RSV-F-F | AACAGATGTAAGCAGCTCCGTTATC |
| RSV-F-R | GATTTTTATTGGATGCTGTACATTT |
| RSV-G-F | CGGCAAACCACAAAGTCACA |
| RSV-G-R | TTCTTGATCTGGCTTGTTGCA |

**Table S3** qPCR reaction conditions

| Stage1 | predenaturation | Rep：1 | 95℃ | 30 sec |
| --- | --- | --- | --- | --- |
| Stage2 | cycle reaction | Reps：40 | 95℃ | 3-10 sec |
|  |  |  | 60℃ | 10-30 sec |
| Stage3 | dissolution curve | Rep：1 | 95℃ | 15 sec |
|  |  |  | 60℃ | 60 sec |
|  |  |  | 95℃ | 15 sec |
